# Interferon-Gamma Release Assay Performance in Pulmonary and Extrapulmonary Tuberculosis

**DOI:** 10.1371/journal.pone.0032652

**Published:** 2012-03-13

**Authors:** Yun Feng, Ni Diao, Lingyun Shao, Jing Wu, Shu Zhang, Jialin Jin, Feifei Wang, Xinhua Weng, Ying Zhang, Wenhong Zhang

**Affiliations:** 1 Department of Infectious Diseases, Huashan Hospital, Fudan University, Shanghai, China; 2 MOH and MOE Key Labratory of Medical Molecular Virology, Shanghai Medical College, Fudan University, Shanghai, China; 3 Department of Molecular Microbiology and Immunology, Bloomberg School of Public Health, Johns Hopkins University, Baltimore, Maryland, United States of America; 4 Institutes of Biomedical Sciences, Fudan University, Shanghai, China; Facultad de Medicina, Uruguay

## Abstract

**Background:**

The diagnosis of tuberculosis remains difficult. This study aimed to assess performance of interferon-gamma release assay (IGRA) in diagnosis of active tuberculosis (ATB) with pulmonary and extrapulmonary involvements, and to determine the diagnostic role of IGRA (T-SPOT.TB) and tuberculin skin test (TST) in BCG-vaccinated population.

**Methods and Findings:**

Two hundred twenty-six ATB suspects were recruited and examined with T-SPOT.TB. Among them, fifty-two and seventy-six subjects were simultaneously tested by TST with 5TU or 1TU of purified protein derivative (PPD). The sensitivity of T-SPOT.TB was 94.7% (71/75), comparable in pulmonary and extrapulmonary disease groups (95.6% *vs.* 93.3%, *P*>0.05), while the specificity was 84.10% (90/107) but differed in two groups (69.2% *vs.* 88.9%, *P* = 0.02). Compared to T-SPOT.TB, TST with 5TU-PPD showed less sensitivity (92.3% vs. 56.4%) and specificity (84.6% vs. 61.5%) (both *P*<0.01); the sensitivity of TST with 1TU-PPD was 27.8%, and despite its specificity identical to T-SPOT.TB (both 82.8%) positive predictive value (PPV) was only 33.3%. By combining T-SPOT.TB with TST (1TU), the specificity rose to 95%, but the PPV stayed unchanged.

**Conclusions:**

IGRA could function as a powerful immunodiagnostic test to explore pulmonary and extrapulmonary TB, while TST failed to play a reliable or auxiliary role in identifying TB disease and infection in the BCG-vaccinated population.

## Introduction

In recent decades, the burden of tuberculosis (TB) has been increasingly falling on developing countries. Although the TB vaccine Bacille Calmette-Guérin (BCG) is broadly vaccinated and DOTS (Directly Observed Treatment, Short-course) programme is well implemented, the incidence rate of active tuberculosis (ATB) in China has been doubled over ten years (39.03/100,000 in 1999 *vs.* 81.09/100,000 in 2009), with the death rate soaring 7-fold in this decade [1]. Despite incorporation of clinical, radiological, pathological and microbiological examinations, diagnosis of ATB can still be difficult. Conclusive diagnostic tests microbial culture and smear for acid-fast bacilli are not sensitive enough to identify all the active cases. Moreover, for extrapulmonary tuberculosis (EPTB), less specific clinical clues can be used and invasive procedures or low bacterial load leads to less chance to establish the pathological or microbiologic diagnosis [2], [3].

Immunoassays capable of detecting the host's immune response specific to TB causative agent *Mycobacterium Tuberculosis* (*M.TB*) has become an alternative diagnostic aid for ATB [2]. Long-time-used tuberculin skin test (TST) has encountered considerable difficulties, mainly due to the disability of its mixed antigens tuberculin purified protein derivative (PPD) to distinguish the true ATB patients from those vaccinated with BCG or sensitized with *Nontuberculous Mycobacteria* (NTM) [4]. Recently, interferon-gamma release assays (IGRAs) have shown their superior diagnostic performance over TST [4], [5], [6], [7], [8], [9] by using at least two specific antigens (ESAT-6 & CFP 10) present exclusively in *M.TB* but absent in BCG strains and most NTM [9], [10]. Herein, we put ELISpot-based-IGRA (T-SPOT.TB) into test to examine how it works especially for identifying EPTB in comparison with pulmonary tuberculosis (PTB). Meanwhile, we compared the performance between IGRA and TST with two currently used doses (5TU; 1TU) to elaborate whether TST is still strong enough to carry on the diagnostic role in ATB for the TB-epidemic and BCG vaccinated populations.

## Methods

This study got ethical approval from Huashan Institutional Review Board (HIRB), the ethics reviewing committee of Huashan Hospital, Fudan University. Informed consent was obtained from all the participants in the written form.

### Study population

A prospective study was conducted in HIV-negative subjects with suspicion of active TB collected between September 2008 and September 2009. A total of 226 patients from China were tested with T-SPOT.TB® at enrollment, together with routine clinical, microbiologic, pathological and radiographic examinations. Individuals were excluded if they have received >30 days of anti-tuberculosis therapy or if they have received the treatment within one year prior to enrollment; those treated for one year or longer before enrollment were otherwise involved. All patients were vaccinated with BCG at early childhood or during adolescence. Major clinical characteristics of recruited subjects were summarized in Table 1.

**Table 1:** Clinical characteristics of 182 patients with suspected active TB.

Characteristics	Total(n = 182)	ATB(n = 75)	No ATB(n = 107)
Age, median (range), yr	52 (14–87)	41(16–84)	51(14–87)
Male/Female	87/95	43/32	44/63
Presence of TB history	13	1	12
Presence of TB contact	11	5	6
TB scar in chest radiographs	23	8	15
Immunocompromised conditions	13	1	12
Liver cirrhosis	1	0	1
Chronic renal failure	2	1	1
Leukemia	2	0	2
Idiopathic myelofibrosis	1	0	1
Hemophagocytic syndrome	1	0	1
Low CD4 count	1	0	1
Immunosuppressive drugs	5	0	5

ATB: active tuberculosis; No ATB: diagnosis other than active tuberculosis.

**Figure 1:**
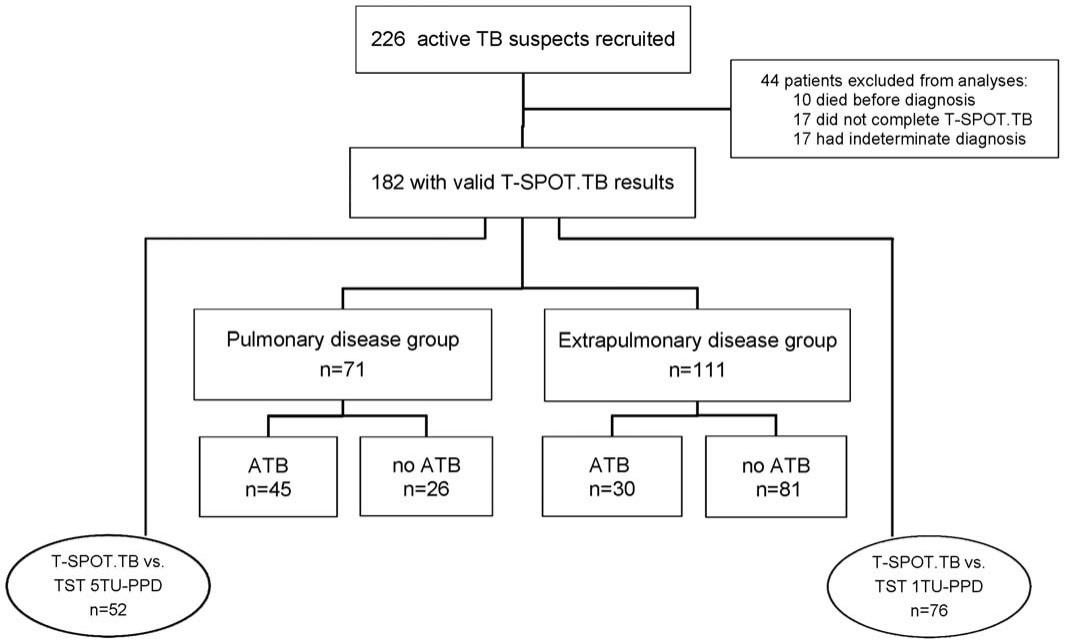
Flowchart of the study population. A total of 226 subjects suspected to have active tuberculosis (ATB) were recruited and 182 were eligible to be included in the final analyses. The analyses were composed of two parts: a study on the diagnostic performance of the T-SPOT.TB on pulmonary and extrapulmonary ATB, and a study comparing the performance between T-SPOT.TB and TST with a dose of 1TU-PPD or 5TU-PPD. ATB, active tuberculosis; no ATB, final diagnosis excluded active tuberculosis.

After a follow-up of at least 3 months, by January 2010, 44 patients were excluded from the study, among which 10 died before final diagnosis, 17 lost follow-up, and 17 had no final diagnosis. The remaining 182 patients were ultimately included for T-SPOT.TB analyses (Figure 1), of which 128 consented to perform TST concurrently, 76 with 1TU-PPD, and 52 with 5TU-PPD.

### Definitions and Diagnosis

TB suspects were defined as patients whose clinical or radiographic manifestations were consistent with active TB [11], but lack of culture or pathological evidence for confirmative diagnosis. Finally, they had one of three diagnoses: (1) ‘culture/biopsy-confirmed ATB’ if final diagnoses were made on the positive culture of *M. TB* from sputum or the presence of caseating granuloma in biopsy specimen; (2) ‘clinical ATB’ if patients, whose clinical presentations were consistent with ATB but lack of bacterial/pathological corroborative evidence, presented manifest clinical or radiographic responses to anti-TB treatment; (3) ‘no ATB’ if the patients did not meet the above two criteria and their clinical presentations diminished spontaneously or following non-TB-related treatment.

### T-SPOT.TB assay

The T-SPOT.TB test was performed following the instructions of the assay kit (Oxford Immunote Ltd., Oxford, UK). Briefly, peripheral blood mononuclear cells (PBMCs) were isolated and incubated with two antigens (ESAT-6 in panel A; CFP-10 in panel B). The procedure was performed in the plates pre-coated with anti-interferon-γ antibodies at 37°C for 16 to 20 hours. After application of alkalinephosphatase-conjugated second antibody and chromogenic substrate, spots were scored using an automated ELISPOT plate reader (AID-Gmb-H, Germany).

### TST

TST was tested on the patients' volar surface of a forearm, by intradermal injection of 1 tuberculin unit (TU) of PPD-S (Statens Seruminstitut, Copenhagen, Denmark) (n = 76) or 5TU of PPD (n = 52). The size of the induration was read at 72 h. Based on the transverse diameter of induration, the cut-off value was determined as follows: induration <10 mm denoted as negative (−); induration ≥10 mm as positive (+).The TST and T-SPOT.TB were all conducted simultaneously.

### Statistical analyses

Sensitivity, specificity, positive predictive value (PPV), negative predictive value (NPV), likelihood ratio positive (LR+), and likelihood ratio negative (LR−) were calculated to evaluate diagnostic performance for the T-SPOT.TB and TST. Ninety-five percent confidence intervals (95%CI) were estimated according to the binomial distribution. Significance was inferred for *P*<0.05. Concordance between the results of TST and T-SPOT.TB was assessed using κ coefficients (κ>0.75, excellent agreement; κ<0.4, poor agreement; 0.75≥κ≥0.4, fair to good agreement). Analyses were performed using statistical software packages (Stata version 9; StataCorp; College Station, TX).

## Results

### Clinical characteristics

Of 182 ATB suspects with valid T-SPOT.TB results, 71 were categorized into pulmonary disease group and 111 in extrapulmonary disease group (Figure 1). Patients with EPTB concurrently with documented PTB were included into PTB group. Eventually, 107 patients excluded ATB and 75 were diagnosed as ATB. Of the latter, 45 were confirmed with culture/biopsy evidences, and 30 as probable ATB cases with clinical evidences. Clinical characteristics of 182 patients are shown in Table 1. The distribution of affected extrapulmonary organs was highly heterogeneous which involved central nervous system, peripheral lymph nodes, pleura, bones, genitourinary system, gastrointestinal tract, and skin (Table 2).

**Table 2:** Comparison of performance of T-SPOT.TB assay in pulmonary and extrapulmonary tuberculosis.

Site of disease	ATB n	No ATB n	T-SPOT.TB(+) n	T-SPOT.TB(−) n	Sensitivity% (95%CI)	Specificity% (95%CI)
Pulmonary disease	45	26	43	18	95.6%(84.9%–99.5%)*	69.2%(48.2%–85.7%)**
Extra-pulmonary disease	30	81	28	72	93.3%(77.9%–99.2%)	88.9%(80.0%–94.8%)
Central nervous system	12	26	12	23	100%	88.5%
Lymphadenitis	3	0	3	0	100%	N/A
Pleurisy disease	2	0	1	0	50%	N/A
Abdominal disease(liver, pancreas, spleen)	6	4	5	4	83.3%	100%
Genitourinary disease	2	6	2	4	100%	66.7%
Bone disease	3	5	3	3	100%	60%
Skin disease	2	6	2	5	100%	83.3%
Other sites	0	33	0	34	N/A	97.1%
Total	75	107	71	90	94.7%(86.9%–98.5%)	84.1%(75.8%–90.5%)

ATB, active tuberculosis; No ATB, diagnosis other than active tuberculosis;

* P>0.05;

** P = 0.017.

### Diagnostic performance of T-SPOT.TB: overall and stratified by disease site

The diagnostic values of T-SPOT.TB for the 182 subjects are presented in Table 3. The overall sensitivity and specificity were 94.70% (95%CI, 86.9%–98.5%), 84.10% (95%CI, 75.8%–90.5%), respectively. There was no significant difference in the sensitivity between the ‘confirmed ATB’ cases (93.3%, 42/45) and the ‘clinical ATB’ cases (96.7%, 29/30; P>0.05). PPV, NPV, LR+ and LR− of the T-SPOT.TB were 80.70%, 95.70%, 5.96 and 0.06, respectively, and the prevalence was 41.2% in the cohort (Table 3).

**Table 3:** Diagnostic performance of T-SPOT.TB assay in 182 active tuberculosis suspects.

Parameter	Value	95%CI
Sensitivity, % (n)	94.70 (71/75)*	86.9–98.5
Specificity, % (n)	84.10 (90/107)	75.8–90.5
PPV, % (n)	80.70 (71/88)	70.9–88.3
NPV, % (n)	95.70 (90/94)	89.5–98.8
LR+	5.96	3.84–9.24
LR−	0.06	0.02–0.17
Prevalence, % (n)	41.2 (75/182)	34.0–48.7

PPV, positive predictive value; NPV, negative predictive value; LR+, likelihood ratio for positive test; LR−, likelihood ratio for negative test.

* The sensitivity for ‘culture/biopsy-confirmed’ subgroup was 93.3% (42/45), with a 95%CI of 81.7%–98.6%; for ‘clinical ATB group’ was 96.7% (29/30), with the 95%CI of 86.8%–99.9%; P>0.05.

The stratified performance by the site of disease is shown in table 2. Among the 71 patients with pulmonary involvement, T-SPOT.TB was positive in 43 of 45 ATB cases, with a sensitivity of 95.6% (95%CI: 84.9%–99.5%) which did not differ significantly from the sensitivity of 93.3% (95%CI: 77.9%–99.2%) in extra-pulmonary disease group (28/30). However, the specificity was 69.2% (95%CI: 48.2%–85.7%) in pulmonary disease group, while a higher specificity was observed in extrapulmonary disease group (88.9%; 95%CI: 80.0%–94.8%; P = 0.017). The results of extrapulmonary disease group were further stratified by affected sites. Notably, apart from pleura (50%; 1/2) and abdomen tuberculosis (83.3%; 5/6), the sensitivity of T-SPOT.TB was as high as 100% for the most affected sites, while the specificity ranged from 60% to 97% (Table 2).

### Risk factors for false-positive outcomes in T-SPOT.TB: overall and stratified by disease site

A number of risk characteristics of patients associated with false-positive and false-negative results were evaluated by multivariate logistic regression. Age≥median age (46-year)’ and ‘history of prior TB’ were turned out to be two independent risk factors related to false-positive outcomes. Odds ratio (OR) between false-positives and true-positives for overall, and in pulmonary and extrapulmonary groups are present individually in Table 4. For the risk factor of ‘age≥median age’, the overall OR was 5.09 (95%CI 1.28–20.25; P = 0.021) whereas 10.71 (95%CI 1.21–94.95; P = 0.009) was obtained in the pulmonary disease group. As for ‘history of prior TB’, OR for overall was 10.06 (95%CI 1.61–62.75; P = 0.013), but OR value could not be calculated in pulmonary group because no false-positive had TB history in this group. By contrast, extrapulmonary disease group showed invalid OR for both risk factors, with 95%CI crossed 1 and P value >0.05 (Table 4). Additionally, No risk factors to false-negatives were elicited.

**Table 4:** Logistic regression analyses of risk factors leading to false-positive results in T-SPOT.TB assay.

	Pulmonary disease			Extrapulmonary disease			Total		
Risk factor	OR	95%CI	P value	OR	95%CI	P value	OR	95%CI	P value
Age≥median age*	10.71	1.21–94.95	0.009	3.13	0.51–19.04	0.216	5.09	1.28–20.25	0.021
History of prior TB	N/A**			4.8	0.35–65.76	0.240	10.06	1.61–62.75	0.013

OR: odds ratio of risk factors between false positive and true positive results.

* Median age: 46 years old, the median age calculated in patients positive for T-SPOT.TB.

** N/A: the value could not be calculated because no false positive subject had TB history in pulmonary disease group.

### Comparisons between T-SPOT.TB and TST with two doses of PPD

In search of the best strategy for active tuberculosis immunodiagnosis, we examined subjects with TST using two PPD strengths: 76 subjects with 1 TU PPD (abbreviated to ‘TST^1TU-PPD^’), and 52 with 5 TU (TST^5TU-PPD^). T-SPOT.TB was simultaneously applied to the two cohorts. The diagnostic values of ‘TST^1TU-PPD^ *vs.* T-SPOT.TB’ and ‘TST^5TU-PPD^ *vs.* T-SPOT.TB’ were listed in Table 5. A statistically significant difference in sensitivity was found between TST^1TU-PPD^ and T-SPOT.TB (27.8% *vs.* 94.4%; P<0.001), but no difference in specificity was observed as they shared the same value (both 82.8%, P = 1.000). The PPV and NPV was 33.3% and 78.7% for TST^1TU-PPD^, and was 63.0% and 98.0% for the T-SPOT.TB, respectively. TST^5TU-PPD^ significantly differed from T-SPOT.TB both in sensitivity (56.4% *vs.* 92.3%, P = 0.001) and specificity (61.5% *vs.* 84.6%, P = 0.0078). The PPV and NPV was 81.5% and 32.0% for TST^5TU-PPD^, and was 94.7% and 78.6% for the T-SPOT.TB, respectively. When the concordance was assessed by kappa coefficient (κ), poor agreement was shown between either comparison, 67.11% for ‘T-SPOT.TB *vs.* TST^1TU-PPD^’ (κ = 0.19) and 63.46% for ‘T-SPOT.TB *vs.* TST^5TU-PPD^’ (κ = 0.28).

**Table 5:** Comparisons of performance between ‘TST^1TU PPD^ vs. T-SPOT.TB’ and ‘TST^5TU PPD^ vs. T-SPOT.TB’.

	TST^1TU PPD^ *vs.* T-SPOT.TB (n = 76)		TST^5TU PPD^ *vs.* T-SPOT.TB (n = 52)	
Parameters	TST^1TU PPD^	T-SPOT.TB	TST^5TU PPD^	T-SPOT.TB
Sensitivity, % (n)	27.8 (5/18)	94.4 (17/18)^a^	56.4 (22/39)	92.3 (36/39)^C^
Specificity, % (n)	82.8 (48/58)	82.8 (48/58)^b^	61.5 (8/13)	84.6 (11/13)^d^
PPV	33.3 (5/15)	63.0 (17/27)	81.5 (22/27)	94.7 (36/38)
NPV	78.7 (48/61)	98.0 (48/49)	32.0 (8/25)	78.6 (11/14)
LR+ (95%CI)	1.61 (0.63–4.10)	5.48 (3.08–9.73)	1.47 (0.70–3.08)	6.00 (1.67–21.54)
LR− (95%CI)	0.87 (0.64–1.19)	0.07 (0.01–0.45)	0.71 (0.41–1.24)	0.09 (0.03–0.28)
Prevalence, %	23.7 (18/76)		75.0 (39/52)	
Concordance,%	67.11		63.46	
Kappa value	0.1866		0.2841	

TST: tuberculin skin test; PPV, positive predictive value; NPV, negative predictive value; LR+, likelihood ratio for positive test; LR−, likelihood ratio for negative test.

a : P = 0.0005;

b : P = 1.000;

c : P = 0.001;

d : P = 0.0078.

### Single and combination diagnostic test efficiency of T-SPOT.TB and TST

**Figure 2:**
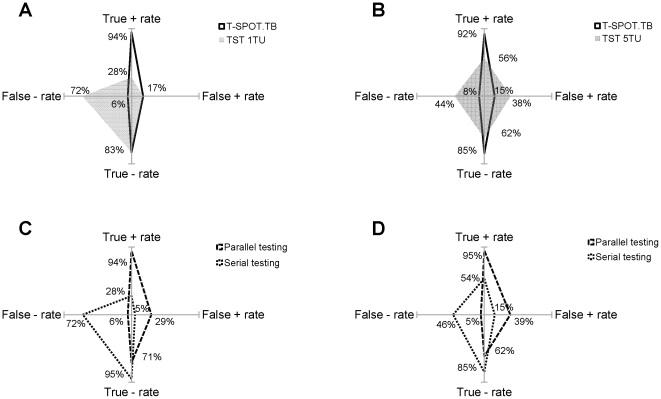
The deviation features of the T-SPOT.TB, TST in single or combination way. The deviation from the gold standard test was compared between T-SPOT.TB and TST^1TU PPD^ (A), T-SPOT.TB and TST^5TU PPD^ (B), and between the combination in parallel and serial way for these two comparisons (C, D). The north, south, east and west poles in each panel represented 100%of the true-positive rate, true-negative rate, false-positive rate, and false-negative rate, respectively, and each observed rate located between the top poles of the axes (100%) and the central origin (0%). In A and B, the shape formed by connecting the diagnostic rates of T-SPOT.TB was outlined by the dark lines and the shape of TST were filled with grey color. In C and D, parallel testing was outlined by dashed lines, and serial testing by dotted lines.

In order to enlarge the features of immunoassays deviating from ‘gold standard’ test, we plotted four basic diagnostic parameters for two comparisons in figure 2, in which a true positive/negative rate line and a false positive/negative rate line were perpendicular to each other, and the shape of a test was formed by connecting its four rates. A ‘gold standard’ test creates a vertical line with 100% true-rates and zero false-rates, and the ‘fatter’ shape a test displays, the more deviation it gets. The most prominent deviation in figure 2A was the false-negative rate (72%) exceeded the true-positive rate (28%) for TST^1TU-PPD^, and the considerable loss of both true-positive and true-negative rates contributed to a nearly symmetric diamond shape for TST^5TU-PPD^ in figure 2B. By contrast, T-SPOT.TB remained a ‘slim’ figure with a slight deviation toward the pole of false-positive in figure 2A and 2B.

We further estimated if diagnosis efficiency could be improved when two tests were combined by parallel and serial testing algorithms. The graphic illustration (Figure 2C and 2D) showed that parallel testing did not bring the sensitivity of T-SPOT.TB up when combining with TST^1TU-PPD^ or it increased from 92% to 95% when combining with TST^5TU-PPD^,but both at the expense of a considerable increase in false-positive rate, resulting in a similar shape as T-SPOT.TB but ‘fatter’ (Figure 2C and 2D). On the other hand, serial testing increased the specificity of T-SPOT.TB from 83% to 95% when combining TST^1TU-PPD^, but did not make any improvement when combing with TST^5TU-PPD^. Meanwhile, the serial PPV was unchanged and decreased by 3.4% in the two combinations respectively (Table 6).

**Table 6:** The effect of parallel and serial testing on sensitivity, specificity and predictive values for T-SPOT.TB and TST in two comparisons.

Test	Sensitivity % (n)	Specificity % (n)	PPV % (n)	NPV % (n)
TST^1TU PPD^	27.8 (5/18)	82.8 (48/58)	33.3 (5/15)	78.7 (48/61)
T-SPOT.TB	94.4 (17/18)	82.8 (48/58)	63.0 (17/27)	98.0 (48/49)
T-SPOT.TB and TST^1TU PPD^ (parallel)^a^	94.4 (17/18)	70.7 (41/58)	50.0 (17/34)	97.6 (41/42)
T-SPOT.TB and TST^1TU PPD^ (serial)^b^	27.8 (5/18)	94.8 (55/58)	62.5 (5/8)	80.9 (55/68)
TST^5TU PPD^	56.4 (22/39)	61.5 (8/13)	81.5 (22/27)	32.0 (8/25)
T-SPOT.TB	92.3 (36/39)	84.6 (11/13)	94.7 (36/38)	78.6 (11/14)
T-SPOT.TB and TST^5TU PPD^ (parallel)^a^	94.9 (37/39)	61.5 (8/13)	88.1 (37/42)	80.0 (8/10)
T-SPOT.TB and TST^5TU PPD^ (serial)^b^	53.8 (21/39)	84.6 (11/13)	91.3 (21/23)	37.9 (11/29)

a , two tests were combined in a ‘parallel’ way that took a positive result when either test was positive and a negative result when both negative.

b , two tests were combined in a ‘serial’ way that took a positive result when both test was positive and a negative result when either negative. The two tests were performed simultaneously and the word ‘serial’ only indicated the combination fashion usually done. PPV, positive predictive value; NPV, negative predictive value.

## Discussion

### Performance characteristics of T-SPOT.TB

Our study revealed a 94.7% overall sensitivity of T-SPOT.TB for detecting ATB which was parallel with our previously published data [12] and within the ranges recently reported elsewhere [8], [13]. Extrapulmonary tuberculosis (EPTB) reported by different countries varies from 15% to 25% in the ATB cases [14]. However, the corroborative epidemiological data for EPTB is rarely available in most TB-endemic country due to diagnostic difficulties. We explored, for the first time, the role of the IGRA in detecting EPTB in Chinese patients. T-SPOT.TB turned out to be sensitive equally in determining PTB and EPTB (95.6% *vs.* 93.3%; P>0.05). In the 111 patients with extrapulmonary involvement, of note was a 100% sensitivity seen in the most investigated sites (Table 2).

Our study revealed a little lower specificity (84.10%) and PPV (80.70%) than most data elicited from the developed countries [8], [13] which may suggest relatively higher prevalence of latent tuberculosis infection (LTBI) in China. For better understanding the causes and likelihood of false-positives in the population tested, we analyzed risk factors leading to false-positives (Table 4). The odds of ‘over-median age’ or ‘having prior TB history’ leading to false-positives was about 5 or 10 times higher than the odds leading to true-positives. Aging and having TB history are known to be the important factors in the individualized clinical risk assessment for LTBI. Similarly, a discrepancy in specificity between pulmonary and extrapulmonary groups (69.2% vs. 88.9%; P = 0.017) can also be explained by the presence of significant difference in OR between groups. OR (10.71) for ‘over-median age’ factor in pulmonary group inferred a nearly 11-fold risk increase caused by the factor to yield a false-positive, whereas the risk factor of age was not related to false-positives in extrapulmonary group.

IGRAs were designed for identification of LTBI and high prevalence of TB may increase the ‘false-positives’ of IGRAs such that a considerable portion of cases with LTBI could be misrecognized as ATB if relying on IGRAs. Moreover, comparing with the specificity, PPV (80.70%) and LR+ (5.96), the sensitivity, NPV (95.7%) and LR− (0.06) strongly indicated IGRAs are better at ruling out ATB than ruling it in. Thus, the real role of IGRAs in high TB-prevalence countries would be appropriate for ruling out a diagnosis of TB.

### Comparing immunodiagnostic strategy for patients with the history of BCG vaccination

The TST suffers from the low specificity and sensitivity when tested on population with high BCG-vaccination coverage and high TB prevalence. TST using a standard dose of 5 tuberculin units (TU) PPD has been widely used, but there is always a disagreement about its role in vaccinated people. The US recommends the interpretation of TST irrespective of prior BCG vaccination, resulting in considerable overdiagnosis of LTBI, while the UK strategy probably misdiagnoses LTBI cases due to the recommendation that the serial TST be contraindicated for BCG-vaccinated persons and IFN-γ testing be used to help interpret positive TST results [15], [16], [17]. For a country having serious TB burden and urgent task to treat active TB, we have to know how much we can rely on this test and how to arrange our best immunodiagnostic strategy.

The study revealed that the sensitivity (56.4%) and specificity (61.5%) for TST with 5TU-PPD (TST^5TU-PPD^) were both beneath the performance of T-SPOT.TB (Table 5) and lower than a roughly 70% sensitivity and 66% specificity reported by two recent comprehensive reviews [8], [18]. Meanwhile, a dose of 1 TU PPD (TST^1TU-PPD^) has long been used to rule in TB cases in some BCG-vaccinated counties like China. Its reliability faces the arguments for its strong specificity and against the weak sensitivity. They were both confirmed by our study (Table 5). Interestingly, a nearly symmetric ‘short’ and ‘fat’ figure for TST^5TU-PPD^ and ‘shot’ in sensitivity but getting ‘fat’ toward false-negative for TST^1TU-PPD^ were graphically presented in figure 2. By contrast, T-SPOT.TB remained ‘thin’ with only a slight growth in false-positive rate presumably reflecting the LTBI prevalence.

TST^1TU-PPD^ showing a specificity numerically identical to the T-SPOT.TB was supposed to identify the presumed LTBI cases like T-SPOT.TB did, and those cases should be simultaneously positive for both tests. However, our observation indicated only 30% of false-positives in one test were also positive for the other. It was very likely that the reasons leading to false-positive for T-SPOT.TB and TST^1TU-PPD^ may not be same: based on our study and other literatures [19], [20], [21], T-SPOT.TB exhibited the ability to detect LTBI, and TST on the other hand was very likely to falsely recognize those vaccinated with BCG or sensitized with NTM even at the small dose of PPD instead of recognizing LTBI. Thus, the dose of 1TU-PPD appeared insensitive to elicit the detectable immune response in people with either TB infection or disease.

We further investigated whether the performance of T-SPOT.TB can be improved by combining with either TST^1TU-PPD^ or TST^5TU-PPD^. A parallel combination test is usually expected to increase sensitivity, , but we found that even at the expense of a big reduction in specificity, the sensitivity of T-SPOT.TB did not increase by parallel combining with TST^1TU-PPD^ (figure 2A and 2C), because the false-negative number upon combination failed to decline (Table 6). Obviously, TST^1TU-PPD^ could not help increase true-negatives by parallel testing due to its low sensitivity. On figure 2B and 2D, parallel and serial testing both displayed a ‘fatter’ feature than T-SPOT.TB with only growth in sensitivity from 92% to 95%, in that TST^5TU-PPD^ helped rule in 1 ATB case (Table 6). The only big improvement in two combination ways was the specificity from 83% to 95% after serial combining the T-SPOT.TB with TST^1TU-PPD^, but along with a paradoxically unchanged PPV. A serial combination is expected to increase specificity and PPV by reducing false positives when both tests must be positive. However, a greater decrement in true-positives than in false-positives after combination suggested that the limitation of TST to identify the true-positives compromised the combinative PPV and made the improved specificity less helpful to determine true active tuberculosis.

There were some noteworthy limitations of this study. Despite no difference in the sensitivity of T-SPOT.TB between the culture/biopsy-confirmed (n = 45) and clinical probable ATB (n = 30) groups, inclusion of those probable cases in the true ATB group may bias the performance for both T-SPOT.TB and TST and their comparisons. Besides, unbalanced factors of age and TB history between comparative groups may also cause study bias, The more subjectives were expected to involve into such investigations.

In conclusion, the TST played a prominent part in the detection of tuberculosis, but its contribution has been on the wane as other immunoassays came into use with superior diagnostic performance over it. This study demonstrated that T-SPOT.TB is a promising tool for diagnosing tuberculosis with pulmonary and extrapulmonary involvement. Moreover, not only cannot TST take major part in immunodiagnosis of ATB, but it was not supported by our study that combining TST with small or regular dose of PPD would become a routine optimized immunodiagnostic strategy in the population with high TB-prevalence and massive BCG-vaccination. We highly recommend that IGRAs should be tested as first priority to diagnose LTBI and ATB for those populations, if applicable.
